# Hypothalamic lipophagy and energetic balance

**DOI:** 10.18632/aging.100393

**Published:** 2011-10-23

**Authors:** Rajat Singh

**Affiliations:** Department of Medicine (Endocrinology) and Molecular Pharmacology, Member of the Diabetes Research Center, Albert Einstein College of Medicine, Bronx, NY 10461 USA

**Keywords:** Hypothalamus autophagy energetic balance

## Abstract

Autophagy is a conserved cellular turnover process that degrades unwanted cytoplasmic material within lysosomes. Through “in bulk” degradation of cytoplasmic proteins and organelles, including lipid droplets, autophagy helps provide an alternative fuel source, in particular, when nutrients are scarce. Recent work demonstrates a role for autophagy in hypothalamic agouti-related peptide (AgRP) neurons in regulation of food intake and energy balance. The induction of autophagy in hypothalamic neurons during starvation mobilizes neuronal neutral lipids to generate neuron-intrinsic free fatty acids that serve to upregulate fasting-induced AgRP levels. Blocking autophagy in AgRP neurons in mice reduces fasting-induced food intake, and increases constitutive levels of anorexigenic hypothalamic proopiomelanocortin and its cleavage product α-melanocyte stimulating hormone. The energetic consequences of these molecular events are decreased body weight and reduced adiposity. The present article discusses this recent finding, as well as considers possible future directions that may help better understand how neuronal autophagy, and its possible reduction during aging, may affect whole body energy balance.

## INTRODUCTION

Autophagy is an evolutionarily conserved cellular turnover process that maintains cellular and energetic homeostasis [1, 2] by eliminating unwanted cytoplasmic debris, for instance aged or dysfunctional organelles and proteins [3]. Two additional lysosomal degradation mechanisms have been characterized in mammalian systems, chaperone-mediated autophagy (CMA) [4] and microautophagy [5]. Although all forms of autophagy function to deliver cytoplasmic substrates into lysosomes, these forms are distinct in their molecular effectors, regulatory elements, and the nature of the cargo delivered to lysosomes [1]. CMA selectively degrades soluble cytosolic proteins displaying a targeting signature [6], the KFERQ motif, which is recognized by a cytosolic chaperone complex that facilitates the delivery of substrates to lysosomes [7]. The binding of the chaperone-protein substrate complex to the lysosome-associated membrane protein (LAMP)-2A allows unfolding and internalization of the protein into lysosomes for degradation [7]. In contrast, microautophagy [5], the least studied form of autophagy, requires the sequestration of cytoplasmic substrates within lysosomal membranes *per se* that are then pinched off to deliver the cargo into the lysosomal lumen. Despite these differences, a common theme in between all these three autophagic pathways is the robust upregulation of these processes during starvation [8].

### Macroautophagy

The orchestration of macroautophagy (henceforth autophagy), a complex and tightly regulated process, requires more than 30 *atg* or autophagy genes as elucidated through elegant yeast genetic studies [9, 10]. Although autophagy occurs at basal levels in all cells, diverse environmental stressors and nutrient deprivation are strong inducers of this degradative machinery [1]. A key negative regulator of autophagy is the nutrient sensor mammalian target of rapamycin (mTOR) [11, 12]. In presence of nutrients, mTOR inhibits autophagy through phosphorylation and inactivation of key downstream targets, unc-51-like kinase1 (ULK1 or Atg1 in yeast), Atg13, and focal adhesion kinase family interacting protein of 200 kD (FIP200), which form part of a complex that initiates autophagy [2, 13, 14]. In contrast, the absence of nutrients inhibits mTOR, allowing ULK1 to form a complex with Atg13 and FIP200 that activates autophagy. Recent studies by a number of independent groups have now shown that a cellular sensor of energy depletion, AMPK, activates autophagy through its ability to phosphorylate and activate its recently elucidated downstream substrate ULK1 [15-17].

**Figure 1:**
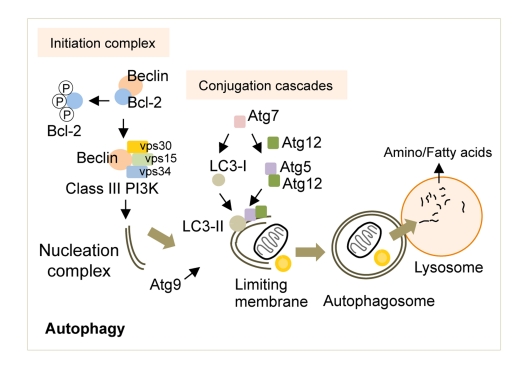
The molecular regulators of autophagy Autophagy induction requires the release of Beclin from Bcl-2, which is then free to form the Class III PI3K that contributes to the formation of the nucleation complex. Two independent conjugation cascades, the LC3-II and the Atg5-12 cascades, serve to elongate the nucleation complex to generate the limiting membrane. The sole transmembrane atg, Atg9, delivers additional membranes for limiting membrane formation. The limiting membrane then sequesters cytosolic cargo and seals upon itself to form an autophagosome. The fusion of autophagosomes to lysosomes results in cargo degradation and release of nutrients into the cytosol. Atg: autophagy gene, LC3: light chain-3, PI3K: phosphoinositide 3-kinase, vps: vacuolar protein sorting.

Briefly, the activation of autophagy in response to nutrient deprivation requires the release of Beclin (Atg6 in yeast) from its binding partner Bcl-2 [18] (Figure 1). Beclin is then free to complex with vacuolar protein sorting (vps) 34, vps15 and Atg14 to form the active class III phosphoinositide 3-kinase (PI3K) complex [19, 20]. The lipid kinase activity of the class III PI3K complex yields phosphatidylinositol 3-phosphate (PI3P) that recruits additional Atg proteins to generate the nucleation complex or the phagophore (in yeast), which gives rise to the autophagosome [2]. Although there is significant debate over the precise cellular locations at which autophagosomes are generated, studies now show that the plasma membrane [21], endoplasmic reticulum [22] or mitochondria [23] may all contribute to *de novo* limiting membrane formation. The elongation of the limiting membrane and autophagosome formation requires two independent conjugation cascades occurring in parallel, the Atg5/12 and the light chain-3 (LC3, Atg8 in yeast) cascades [24-26], both of which require a number of additional Atg proteins including the crucial ubiquitin E1-like ligase, Atg7 [27]. In addition, the shuttling of Atg9, the exclusive transmembrane Atg, to the site of autophagosome formation helps provide membranes for the elongation of the limiting membrane [28]. The autophagosomes then sequester cytoplasmic material destined for degradation and deliver these to lysosomes by fusing with them where acidic hydrolases serve to breakdown cargo. The broken down products, amino acids and fatty acids are then reutilized following their efflux in the cytosol though lysosomal membrane permeases and transporters.

### Lipophagy

Although autophagy degrades cellular cargo in an “in-bulk” manner, this pathway demonstrates significant selectivity for the degradation of mitochondria, endoplasmic reticulum, ribosomes, and peroxisomes through processes termed mitophagy [29], reticulophagy [30], ribophagy [31], and pexophagy [32], respectively. The recent elucidation of a role for autophagy in degrading lipid droplets by lipophagy introduced autophagy as a key regulator of cellular lipid metabolism [1, 33, 34]. In fact, an acute exposure of cultured hepatocytes to distinct lipid stimuli, for instance fatty acids or culturing in methionine and choline-deficient medium, was sufficient to induce autophagy [33]. The induction of autophagy led to the breakdown of hepatocellular lipid droplets to increase availability of free fatty acids for β-oxidation [33]. The genetic or pharmacological inhibition of autophagy increased hepatocellular lipid droplet content, as determined by fluorescent BODIPY labeling, electron microscopic analyses, and by the biochemical determination of triglyceride levels [33]. Triglyceride accumulation in autophagy-deficient cells did not occur from increased synthesis but was a consequence of reduced mobilization of lipid droplets. A testament of this was the detection of the autophagosome marker LC3-II within lipid droplet fractions from livers of starved rodents, and that liver-specific deletion of the autophagy gene, *atg7*, increased lipid droplet number and size as revealed by oil red O staining [33].

While acute lipid loading has been shown to activate autophagy, chronic lipid exposure such as high fat feeding displays an inhibitory effect on autophagy in the liver [33] by reducing autophagosome-lysosomal fusion [35]. Additionally, lipophagy has now been shown to function in a number of diverse cell types such as mouse embryonic fibroblasts [33], and in distinct neuronal populations, for instance primary hypothalamic [36], and striatal neurons [37]. In fact, in a recent study in primary striatal neurons [37], mutations in huntingtin that impaired recognition of cytoplasmic cargo including lipid droplets led to the remarkable increase in neuronal lipid accumulation. These exciting developments demonstrate that autophagic sequestration and lysosomal degradation of cellular lipid stores may be a generalized mechanism for the mobilization of cytosolic lipid stores [38].

### Lipophagy in hypothalamic neurons and energy balance

The mediobasal hypothalamus (MBH) consists of distinct neuronal populations [39], the agouti-related peptide (AgRP) and the proopiomelanocortin (POMC) neurons, which form part of a complex neural circuit that integrates nutritional, hormonal and neural information to regulate food intake and energy metabolism [40]. The AgRP neurons secrete orexigenic AgRP that increase food intake by serving as a physiological antagonist for the melanocortin receptors on anorexigenic POMC neurons [41]. In addition, the AgRP neurons provide inhibitory γ-aminobutyrate (GABA)-ergic projections on to POMC neurons [42]. In contrast, POMC neurons express the POMC precursor that undergoes significant processing to generate α-melanocyte stimulating hormone (MSH), which curtails appetite and promotes peripheral energy expenditure [43].

Although neuronal mechanisms that regulate food intake and energy balance are yet unclear, a number of candidate signaling molecules have been shown to contribute significantly to the control of appetite [44]. Studies have demonstrated crucial roles for hypothalamic mTOR [45], AMPK [46, 47], PI3K [48, 49], FoXO1 [50, 51] and insulin signaling cascade in the regulation of food intake and energy balance [52, 53], and intriguingly, all of these signaling pathways have been shown to converge upon autophagy [12, 15-17, 54]. Apart from these pathways, levels of neuronal metabolites, such as free fatty acids have also been shown to modulate food intake [44]. For instance, mitochondrial entry and oxidation of free fatty acids regulated via the carnitine palmitoyltransferase (CPT) 1-dependent pathway has been associated to appetite regulation. Activation of central AMPK in response to elevated cellular AMP/ATP ratio results in the phosphorylation and inactivation of acyl CoA carboxylase that, in turn, reduces production of malonyl CoA, the allosteric inhibitor of CPT1 [55, 56]. The depletion of malonyl CoA removes its allosteric inhibition on CPT1 and facilitates mitochondrial fatty acid entry, oxidation and increased ATP production required for neuronal activation. In fact, findings from a recent study suggest that the availability of neuron-intrinsic free fatty acids and its metabolism may provide the bioenergetic requirements for hypothalamic neuronal activation and firing [56]. Although neuron-intrinsic free fatty acids may be necessary for the initiation of food-seeking behavior, the mechanisms that regulate neuronal availability of free fatty acids are unclear.

Since hypothalamic activity of the autophagy regulator, mTOR, controls appetite and energy balance [45], and given the consideration that hypothalamic neuron-intrinsic free fatty acids regulate food intake; we asked whether induction of autophagy in AgRP neurons during nutrient deprivation increased neuronal free fatty acid availability that activated feeding mechanisms. Our studies demonstrate that starvation, indeed, induced autophagy within the AgRP-expressing hypothalamic GT1-7 cells and in the MBH [36]. In parallel with our earlier findings in cultured hepatocytes, we observed that the provision of an acute lipid stimulus to cultured hypothalamic cells markedly increased autophagic flux (increased delivery of autophagic substrates to lysosomes), and increased protein degradation via the lysosomal system [36]. Interestingly, exposure to free fatty acids increased hypothalamic levels of phosphorylated AMPK and ULK1 [36], which have recently been shown to activate autophagy [15-17]. These findings raise fundamental questions regarding a role for hypothalamic AMPK as part of a neuronal fatty acid sensing apparatus that eventually converges upon and activates autophagy. Starvation typically increases circulating free fatty acids, and thus, we asked whether the source of increased hypothalamic lipids during starvation is the periphery, as suggested in an earlier study [56]. Indeed, culturing hypothalamic cells in serum from starved rodents increased neuronal lipid accumulation when compared to cells exposed to serum from fed rodents. Consistent with these observations were the findings of increased fatty acid uptake and triglyceride synthesis in hypothalamic cells subjected to serum removal, and in MBH explants from food-restricted mice [36]. Interestingly, the immediate fate of free fatty acids taken up by hypothalamic cells was triglyceride (TG) synthesis since exposure of starved cells to triacsin C, an inhibitor of TG synthesis, completely blocked TG synthesis during starvation [36]. The fact that fatty acids are rapidly esterified to TG within lipid droplets underscores the requirement of a lipolytic mechanism that breaks down lipid droplets to increase availability of cell-intrinsic free fatty acids during starvation. Indeed, treatment of cultured hypothalamic cells and primary hypothalamic neurons to oleic acid increased interactions between phagosomes/lysosomes and neuronal lipids, demonstrating that as we observed in liver, hypothalamic autophagy functions to deliver neuronal lipid droplets to lysosomes [36]. The functional significance of these interactions was generation of neuronal free fatty acids, since blocking lysosomal degradation or silencing *atg5*, a second autophagy gene, in hypothalamic cells reduced cellular free fatty acid levels in response to starvation [36]. In addition, the inhibition of lysosomal function reduced the increase in AgRP levels that occurred in response to fatty acid treatment, following culture in fatty acid-enriched starved rodent serum, in response to serum deprivation, and even in primary hypothalamic neurons cultured under basal conditions [36]. Furthermore, inhibiting AgRP neuron-selective autophagy *in vivo* reduced MBH AgRP levels and food intake specifically in response to fasting [36], although no differences in AgRP levels or food intake were observed between controls and the autophagy-null mice under basal fed conditions. These *in vivo* findings led us to examine the mechanism for reduced adiposity in the AgRP neuronal autophagy-deficient rodents. Indeed, deleting *atg7* in AgRP neurons increased hypothalamic levels of the POMC precursor and its cleavage product α-MSH that, in turn, reduced body weight and adiposity by increasing locomotor activity and upregulating levels of the adipose triglyceride lipase in epididymal fat pads [36].

**Figure 2:**
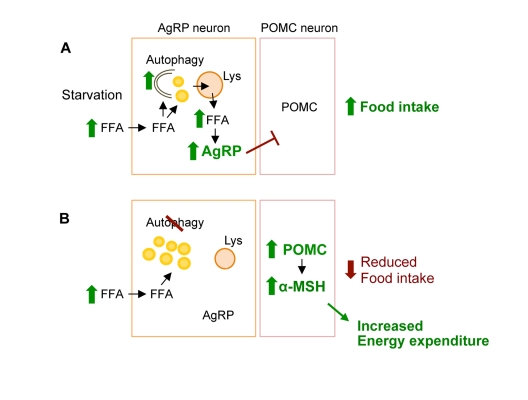
Conceptual framework for role of hypothalamic autophagy in food intake and energy balance. (**A**) During starvation hypothalamic uptake of increased circulating free fatty acids (FFA) leads to induction of neuronal autophagy. The immediate fate of the FFA is triglyceride synthesis within neuronal lipid droplet (LD). Activated autophagy breaks down LDs to generate neuron-intrinsic FFA that increase AgRP expression to promote food intake. (**B**) Genetic and/or pharmacological inhibition of autophagic degradation of lipids, lipophagy, leads to increased neuronal LDs, and reduced levels of FFA and AgRP in response to starvation. Blocking autophagy in AgRP neurons *in vivo* increased hypothalamic levels of POMC and its cleavage product α-MSH that contributed to decreased food intake in response to fasting, and increased peripheral energy expenditure.

Based on these observations, we now provide a conceptual framework for considering how induction of autophagy during starvation functions to mobilize neuronal lipids for the controlled availability of neuron-intrinsic free fatty acids, which upregulate AgRP levels and increase food intake specifically in response to starvation (Figure 2) [36].

### Aging, autophagy, and future directions

Although our studies reveal a new role for autophagy in hypothalamic AgRP neurons in control of food intake and energy balance [36], there are several questions that will need to be addressed in future studies. For instance, is the function of AgRP neuronal autophagy in control of food intake limited to lipid mobilization and generation of free fatty acids, or are there additional roles for autophagy in processes as diverse as actin remodeling or regulating cell shape that might be crucial for synaptic plasticity, neuron activation and firing. While autophagy-regulated free fatty acid availability drives expression of AgRP, autophagy might also regulate levels of key neuronal proteins through controlled lysosomal degradation that may modulate appetite and/or energy balance. Additional questions directly relevant to the present work include mechanisms into how free fatty acids increase AgRP levels. Detailed work from Kitamura and colleagues highlight roles for FoxO1-dependent mechanisms in control of AgRP expression [51], which begs the question whether autophagy-derived free fatty acids might possibly feed on to these signaling cascades to drive AgRP levels during starvation. Additionally, neuronal free fatty acids might form secondary lipid species that, in turn, may possibly drive PPAR-dependent transcriptional events within the hypothalamus to modulate appetite [57].

Since the AgRP and POMC neuronal network coordinate the regulation of food intake, the next obvious question pertains to roles for autophagy in the hypothalamic POMC neuronal population? POMC neurons in the MBH positively regulate energy expenditure in the periphery, thus it could be speculated that reduced autophagy in these neurons may contribute to an adiposity phenotype. Indeed, unpublished results from our laboratory reveal that genetic ablation of autophagy selectively in hypothalamic POMC neuron promotes an early onset adiposity and altered glucose homeostasis. Additionally, a recent study that blocked hypothalamic autophagy using acute lentiviral-mediated silencing of *atg7* in the MBH has revealed a marked increase in adiposity and reduced glucose tolerance in the absence of hypothalamic autophagy [58]. These results indicate that the predominant effect of blocking autophagy in all cell types in the MBH, as would be expected from use of this experimental approach, is increased adiposity occurring possibly from the masking of the beneficial affects observed from the singular targeting of autophagy in AgRP neurons [36].

Autophagy is typically considered to decrease with age [59, 60], and aging alters energetic balance and body fat distribution [61], in addition to affecting food intake [62], although marked reduction in food intake is more commonly observed in advanced stages due to terminal conditions. Thus, it is not surprising that redundant central mechanisms may be involved in control of food intake that occurs under basal conditions, and that loss of one mechanism may allow additional processes to compensate, which might explain why appetite *per se* may not decline until very late in life. Elegant work from Andrews and colleagues shows that AgRP/NPY neurons may be protected against reactive oxygen species (ROS) via uncoupling protein (UCP) 2-dependent mechanisms [56], and these results have led to the speculation that progressive POMC neuronal damage from higher basal ROS levels observed in these neurons may contribute to obesity-related disorders observed during aging [56]. In parallel with this line of thought, one might postulate that the activity of POMC neuronal autophagy may decrease relatively early in the aging process, as opposed to autophagy in AgRP neurons, which might explain the progressive increases in adiposity occurring during middle age, although food intake may not decline until very late in life.

A number of interventions, nutritional and/or pharmacological, have been shown to prevent the decline of autophagy observed during aging. Calorie restriction is a strong inducer of autophagy, and has been shown to promote lifespan extension in a number of species [63-65]. In fact, *Caenorhabditis elegans* lacking autophagy genes did not display extended longevity in response to caloric restriction [66, 67]. Calorie restriction also delayed the incidence of diabetes, cancer, cardiovascular disease and brain atrophy, as well as mortality in primates [68], although it was not shown whether these effects in primates were mediated through upregulation of autophagy. Although benefits of calorie restriction are thought to be exerted, at least in part, via reduction of visceral adiposity [69, 70], it would be immensely interesting to test whether calorie restriction may restore some of these peripheral metabolic defects through direct effects on autophagy in the central nervous system. Restricting nutrients downregulate mTOR, and hyperactive mTOR has been demonstrated to bring about a number of ill effects associated with the aging process, for instance generation of cellular senescence *in vitro* [71-73], dysregulated protein synthesis and its effects on proteotoxicity [74], as well as reducing insulin sensitivity through S6K1-mediated degradation of IRS1/2 [75]; therefore favorable effects of calorie restriction may be attributed to both reduced mTOR activity and/or improved cellular quality control via the upregulation of autophagy [76, 77]. Pharmacological interventions that upregulate autophagy, such as use of rapamycin that inhibits the negative regulator of autophagy, mTOR, have been shown to confer longevity in yeast [78], worms [66], and in aged heterogeneous mice [79]. Additional pharmacological agents, in particular spermidine [80], have been shown to mediate lifespan extension through the upregulation of autophagy [81]. Although, these agents may have contributed to lifespan extension through upregulation of autophagy in peripheral tissues, it may be expected that pharmacological upregulation of autophagy in the central nervous system using one or more of these agents will protect against adiposity and reduced insulin sensitivity in peripheral tissues observed during aging.

Indeed, if there is a case for restoration of autophagy during aging, then the question that needs to be addressed first is what might be the mechanism for reduced autophagy during aging? We [33], and others [35, 82] have shown that prolonged high fat feeding inhibits autophagy in the liver, which then sets up a vicious circle that furthers lipid accumulation. Aging is typically associated with lipid accumulation, and although the dynamics and consequences of lipid accumulation during aging may be quite distinct from those observed with high fat feeding, it is possible that aging-induced chronic lipid build up in neurons may dysregulate autophagy and energetic balance through one of many effects including generation of reactive oxygen species, changes in membrane lipid composition and function, and hyperactivation of key upstream regulators, for instance mTOR. A recent study highlights a highly complex neuroendocrine modulation of autophagy by the adiposity hormone leptin, suggesting the presence of yet uncovered links between adiposity hormones, autophagy, the biology of aging, peripheral insulin signaling and energy balance [83]. Moreover, the functional consequences of reduced autophagy in nutrient sensing neurons need to be fully characterized. The further characterization of roles for autophagy in distinct neuronal subsets, as well as mechanistic insights into how autophagy modulates neuron function to affect whole body energetic balance may have implications for development of novel therapeutic strategies in the fight against obesity and insulin resistance.
